# TASC (Telehealth After Stroke Care): a study protocol for a randomized controlled feasibility trial of telehealth-enabled multidisciplinary stroke care in an underserved urban setting

**DOI:** 10.1186/s40814-022-01025-z

**Published:** 2022-04-11

**Authors:** Imama A. Naqvi, Ying Kuen Cheung, Kevin Strobino, Hanlin Li, Sarah E. Tom, Zehra Husaini, Olajide A. Williams, Randolph S. Marshall, Adriana Arcia, Ian M. Kronish, Mitchell S. V. Elkind

**Affiliations:** 1 Department of Neurology, Columbia University Vagelos College of Physicians and Surgeons, New York, NY USA; 2 Division of Stroke and Cerebrovascular Diseases, Columbia University Medical Center, 710 West 168th Street, New York, NY 10032 USA; 3 Department of Biostatistics, Mailman School of Public Health, Columbia University, New York, NY USA; 4 Vagelos College of Physicians and Surgeons, Columbia University, New York, NY USA; 5 NewYork-Presbyterian Hospital, New York, NY USA; 6 Department of Neurology Vagelos College of Physicians and Surgeons and Department of Epidemiology, Mailman School of Public Health, Columbia University, New York, NY USA; 7 University of Michigan, Ann Arbor, MI USA; 8 Columbia University School of Nursing, New York, NY USA; 9 Center for Behavioral Cardiovascular Health, Columbia University Irving Medical Center, New York, NY USA

**Keywords:** StrokeHypertension, Multidisciplinary, Telehealth, Disparities, Self-efficacy

## Abstract

**Background:**

Hypertension is the most important modifiable risk factor for recurrent stroke, and blood pressure (BP) reduction is associated with decreased risk of stroke recurrence. However, hypertension remains poorly controlled in many stroke survivors. Black and Hispanic patients have a higher prevalence of uncontrolled BP and higher rates of stroke. Limited access to care contributes to challenges in post-stroke care. Telehealth After Stroke Care (TASC) is a telehealth intervention that integrates remote BP monitoring (RBPM) including nursing telephone support, tailored BP infographics and telehealth video visits with a multidisciplinary team approach including pharmacy to improve post-stroke care and reduce stroke disparities.

**Methods:**

In this pilot trial, 50 acute stroke patients with hypertension will be screened for inclusion prior to hospital discharge and randomized to usual care or TASC. Usual care patients will be seen by a primary care nurse practitioner at 1–2 weeks and a stroke neurologist at 1 and 3 months. In addition to these usual care visits, TASC intervention patients will see a pharmacist at 4 and 8 weeks and will be enrolled in RBPM consisting of home BP monitoring with interval calls by a centralized team of telehealth nurses. As part of RBPM, TASC patients will be provided with a home BP monitoring device and electronic tablet that wirelessly transmits home BP data to the electronic health record. They will also receive tailored BP infographics that help explain their BP readings. The primary outcome will be feasibility including recruitment, adherence to at least one video visit and retention rates. The clinical outcome for consideration in a subsequent trial will be within-patient change in BP from baseline to 3 months after discharge. Secondary outcomes will be medication adherence self-efficacy and satisfaction with post-stroke telehealth, both measured at 3 months. Additional patient reported outcomes will include depression, cognitive function, and socioeconomic determinants. Multidisciplinary team competency and fidelity measures will also be assessed.

**Conclusions:**

Integrated team-based interventions may improve BP control and reduce racial/ethnic disparities in post-stroke care. TASC is a post-acute stroke care model that is novel in providing RBPM with tailored infographics, and a multidisciplinary team approach including pharmacy. Our pilot will determine if such an approach is feasible and effective in enhancing post-stroke BP control and promoting self-efficacy.

**Trial registration:**

ClinicalTrials.gov NCT04640519

**Supplementary Information:**

The online version contains supplementary material available at 10.1186/s40814-022-01025-z.

## Background

Improvements in stroke prevention, acute treatment, and organized systems of care for acute stroke have contributed to declines in stroke mortality observed over the past decade. With increasing survival rates after stroke and expected increases in stroke related to population aging, however, the prevalence of stroke is projected to increase by 3.4 million by 2030 [1–3]. Despite these projections, there has been little research on improving post-acute care models for stroke survivors.

Stroke incidence, mortality, and recurrence vary by race/ethnicity [1, 4]. Non-Hispanic Black, Hispanic, and Native Americans have a higher risk of stroke than non-Hispanic White (NHW) Americans [5–9]. Projected increases in stroke prevalence also vary by racial and ethnic category, with the largest rises expected in Hispanic and Black men and women race-ethnicity [2]. The Hispanic Paradox is the recognition that although Hispanics are burdened with increased risk factors, comorbid conditions, and limited access to health care, they have lower case fatality rates than NHWs for many diseases, including stroke. However, the survival advantage after ischemic stroke appears to have recently disappeared [3, 10]. Systems of care for stroke survivors must be developed with mechanisms to address racial/ethnic disparities in stroke outcomes.

Hypertension is the most important risk factor for first stroke occurrence [11], and reduction in blood pressure (BP) after stroke is associated with reduced risk of stroke recurrence [12–14]. Small reductions in systolic BP after stroke (5 mmHg) are associated with greater than 20% reduction in recurrent stroke risk [15]. However, hypertension remains poorly controlled after an incident stroke in up to 55% of survivors [16–18]. Less is known about BP control rates in Hispanic patients after stroke, but they are known to have lower BP control rates compared to White patients in the general population [19]. Race/ethnicity, lower socioeconomic status, medication adherence, self-efficacy, marital status, and level of independence have been associated with poor BP control [16, 18, 20–22].

Efforts to reduce ethnic and racial disparities in BP control among stroke survivors have not been effective [23]. At the patient level, adherence to antihypertensive medications is poor among stroke patients, with up to a third reporting non-adherence [24]. There is also system mistrust and limited disease awareness among patients in these groups. At the provider level, there is also poor adherence to guidelines when it comes to care for Black patients. Even when there is adherence to guidelines at the time of discharge, there is clinical inertia in the subsequent management of hypertension that impacts BP control. At the health care systems level, Black and Hispanic stroke patients have poorer access to care, and medications compared to White Americans as a result of differences in health insurance coverage [4]. These factors contribute to challenges in developing system-level interventions for post-stroke care, including BP control, amplified in underserved minorities. Interventions that seek to improve post-stroke BP control methods and expand access to post-stroke care are needed to effectively address these disparities by reducing detrimental therapeutic inertia by clinicians and improving self-efficacy for hypertension management in patients.

Among stroke patients, isolated behavioral and educational interventions have not been shown to impact BP control [19]. Organizational interventions that incorporate multidisciplinary teams and integrated care service with quality management, well-suited to address multiple barriers, have been shown to produce improvements in BP control in the general population [25–29]. Less is known about their impact on BP control after stroke. In particular, integrated care with pharmacy-guided support to address barriers to medication adherence has been shown to augment BP control in hypertensive patients, but also has limited evidence among stroke patients [27].

Telemedicine has been implemented successfully for acute stroke care and improves access to large stroke centers; but the impact of telemedicine on racial and ethnic disparities, particularly for secondary stroke prevention, remains understudied in acute stroke systems of care [30]. Tele-neurology visits have been shown to improve access to general neurologists in rural areas for outpatient clinical follow ups [31]. The coronavirus disease 2019 pandemic has accelerated the use of telemedicine [32]. Rapid transition to virtual visits for outpatient care among patients with neurological diseases has been implemented during the pandemic, allowing patients to communicate via smartphones and other devices. As telehealth services expand, there may be incentivization to assemble infrastructures at the health policy level. Telemedicine has the potential to increase disparities in usage of audio-video technology among vulnerable populations, particularly among older age groups, and patients with public insurance [33]. However, internet accessibility and usability has been noted in a majority of stroke survivors and their informal caregivers [34]. Hence, this suggests the need to rigorously develop and test telehealth-based post-stroke interventions with focused attention on disparities.

Telemonitoring have shown improvements in BP control in the general population [35–37]. Successful interventions include those that incorporate a team-based approach to care and those that utilize telehealth and home BP monitoring [36–43]. Among stroke patients, isolated behavioral and educational interventions have not been shown to impact BP control [44]. More recently, community-based efforts have been successfully underway to address disparities among Black and Hispanics with home BP telemonitoring and nurse care management [45]. Moreover, a randomized pragmatic trial evaluating real-world clinical practice model showed a trend toward successful home BP monitoring, but was unable to be consistently incorporated with only 35% of patients attending clinic visits for follow up management [46].

### Conceptual framework

Following a stroke, many patients have multiple barriers to optimizing care, including limited access to outpatient care due to impaired mobility, poor access to transport, and lack of practical social support. We concur with the prevailing idea that effective secondary prevention interventions should be multi-faceted to address the complex medical and social needs of stroke patients [23]. To conceptualize the care of complex stroke patients, we utilize the theoretical framework of the cumulative complexity model [47]. The cumulative complexity model (CCM) is a patient-centered model in which imbalances in patient capacity (physical/mental functioning, fatigue, finances, literacy, and social support) and patient workload of demands such as work responsibilities, self-care, and scheduling appointments/transportation can lead to poor outcomes. The focus in this model is on patient complexity, instead of complex interventions or outcomes, to emphasize factors at the patient level including care delivery including access and utilization to lead to good outcomes. In organizing a system of outpatient stroke care, all aspects of the patient workload-capacity must be addressed. Reducing barriers to health care access and strengthening patient self-management is vital.

Integration is key to health care reform and principles identified suggest a collective approach to positively impact patient, provider, and system outcomes to support health integration and help gauge feasibility [48]. These include patient-focused interprofessional team efforts combined with health information technology to increase access while building organizational systems of care.

Acute stroke patients returning home not only have reduced capacity but also increased workload of demands. Post-acute stroke period is a particularly vulnerable time for patients with increased risk of stroke recurrence and transitions of care from the inpatient setting. Uncontrolled hypertension is a major risk factor for stroke recurrence and is particularly difficult to control after an acute stroke [49]. Transitional care is careful “bridging” between quality hospital care to outpatient care. A nurse practitioner focused care model, Transitional Stroke Clinic (TRACS) in North Carolina was able to demonstrate 50% decrease in 30-day readmission with post-discharge calls and early follow up [50].

Pharmacist-guided care has been found to improve patients’ BP control. They have also contributed to related interventions including medication counseling, medication reconciliation, or follow up, recommending initiation of therapy and preventing adverse drug reactions [27, 51–53]. Prescription and medication reconciliation errors during transitions of care are common, and sole responsibility of the pharmacist may not prevent medication errors [54]. Simple measures such as using mail order pharmacy instead of local pharmacy have showed improvement in medication adherence in the stroke population [55]. Although not generalizable to a diverse population, a more recent cluster-randomized trial in a primary care clinic group found home BP telemonitoring with pharmacist management to effectively lower BP and cardiovascular outcomes, as well as costs over 5-year follow up [56]. Moreover, pharmacist’s role in team-based care with medication titration has been found to be most effective compared to physician care and multilevel strategies without team-based care with an impact on BP control and cost savings [29, 57]. However, literature among stroke patients is limited by lack of robust and focused studies in multidisciplinary collaboration as well as the role in promoting self-efficacy [51].

Particularly among vulnerable populations, affording home BP devices may not be possible on a limited income. Home BP monitoring by itself without access to technology and ability to transmit data through limited internet access may be another hindrance. Patients may be unable to measure BP accurately without additional support. RBPM designed to be inclusive BP monitoring kit and electronic devices with remote nursing assistance for measurements and severely elevated readings overcome these barriers to BP monitoring among stroke patients returning home.

Visual aids such as tailored infographics, in the form of information-rich contextualized information, may also be important to motivate health promoting behaviors. The use of tailored infographics is an innovative, emerging approach to supporting comprehension of personal health information among patients with varying levels of health literacy. As visually communicated health information, they are a means to make medical information more accessible, and to enhance patient’s decision-making capabilities regarding their health [58]. Developed with local community participatory design, these effectively engage patients as clinical communication tools to drive self-health management. They may be most effective in building knowledge through to improvement in patient reported outcomes [59]. The goal is to improve outcomes by empowering self-management of hypertension and by providing accessible, integrated services and educational materials while considering sociodemographic health determinants.

For post-stroke care, the feasibility, and outcomes of multidisciplinary remote BP monitoring with tailored infographics, and multidisciplinary telehealth visits inclusive of pharmacists have not been studied in healthcare systems serving the highest-risk populations, including urban underserved communities. This proposed study is designed to assess the feasibility of a telehealth-based multidisciplinary approach that incorporates remote BP monitoring as an innovative approach to post-stroke care that reduces disparities in stroke outcomes in an underserved, urban setting.

### Study aims and objectives

The study aims to explore the feasibility of the TASC intervention and trial methodology.

#### Primary aims

**Fig. 1:**
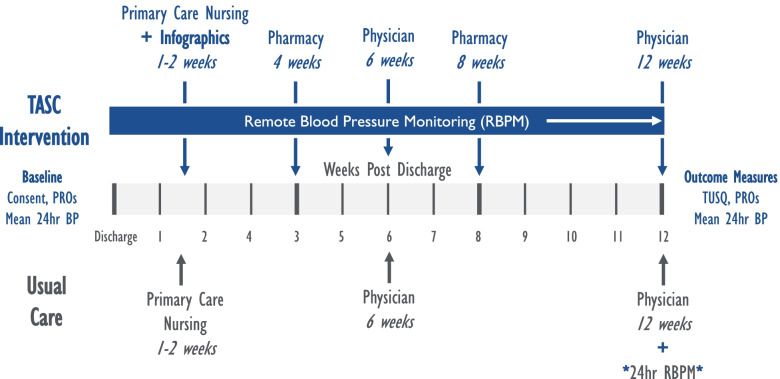
Telehealth after stroke care (TASC): multidisciplinary access with remote BP monitoring intervention versus usual care

To develop and establish feasibility of the TASC model (an integrated telehealth approach to blood pressure management after stroke, Fig. 1).

#### Secondary aims

To explore the effect of the TASC intervention on patient-reported outcomes including self-efficacy in medication adherence and socioeconomic determinants.

Hypothesis: Remote BP monitoring and telehealth visits with a multidisciplinary approach including tailored infographics, primary care, stroke specialist, and pharmacy visits will enhance BP control and promote self-efficacy compared to a multidisciplinary approach that includes primary care and stroke specialist visits, alone.

Ultimately, feasibility metrics and patient feedback through validated assessments from study participants, in addition to interdisciplinary team competencies, will be used to refine the intervention and inform the development of a protocol for a further definitive trial in which the effectiveness and cost-effectiveness of TASC will be assessed.

## Methods

### Study design and overview

This is a parallel two-armed feasibility randomized controlled trial comparing Telehealth After Stroke Care (TASC) intervention (multidisciplinary team care inclusive of pharmacists with home BP monitoring with centralized remote nursing call support and tailored infographics to facilitate understanding of BP data) with usual care. It aims to evaluate the feasibility of a telehealth-based model providing multidisciplinary access including nursing, pharmacy, and physician care, and assess feasibility of an integrated telehealth approach to blood pressure management after stroke. The outcome of BP control will be defined by change in mean awake systolic blood pressure from baseline at the time of discharge through remote monitoring at 3 months.

**Fig. 2:**
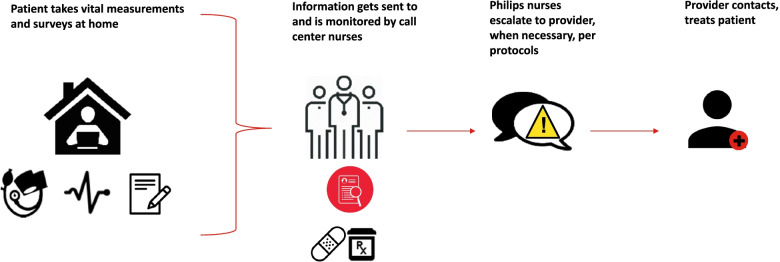
Remote BP patient monitoring overview of system workflow

Remote BP monitoring will be provided with communication through tailored electronic tablet devices with feedback to centralized nurses for tailored telephone-based counseling and communication to providers as needed for elevated readings (Fig. 2).

We will randomize 25 patients to the TASC intervention, including remote blood pressure monitoring, and 25 patients to usual care. Feasibility measures of interdisciplinary team competency, recruitment, retention, fidelity of implementation, and telehealth patient satisfaction surveys will be assessed in the intervention arm.

To our knowledge, no studies providing accessible integrated care including telehealth and remote BP monitoring with multidisciplinary teams including pharmacy and nursing have been conducted. Such an intervention may reduce racial disparities in post-stroke BP control by providing access to care and facilitating self-efficacy to improve health outcomes.

### Setting and sample

Patients will be enrolled from the Columbia University Irving Medical Center (CUIMC) Comprehensive Stroke Center in the underserved area of Northern Manhattan that serves a majority Hispanic low-income community, as the representative Northern Manhattan Study (NOMAS) cohort reports a 53% Hispanic race/ethnicity with 44% on Medicaid/no insurance [60]. The CUIMC Comprehensive Stroke Center refers patients post-discharge with Medicare/Medicaid to the Ambulatory Care Network (ACN) stroke clinic. Our hospital’s ACN, serving a predominantly Hispanic, and Black low-income urban population, started to provide telehealth general neurology visits with tele-pharmacy support to identify gaps in medication education and reconciliation before the coronavirus pandemic [61].

### Eligibility criteria

Eligible patients would include those with an acute ischemic or hemorrhagic stroke that are discharged home after hospitalization (Table 1). Hospital and clinic records contain other data including patient demographics and medical information. Efforts will be made to collect social determinants of health through validated measures for financial, food, and housing insecurity along with demographics including income, education, and housing information. Questions regarding internet use and access to electronics will also be asked in addition.

**Table 1:**
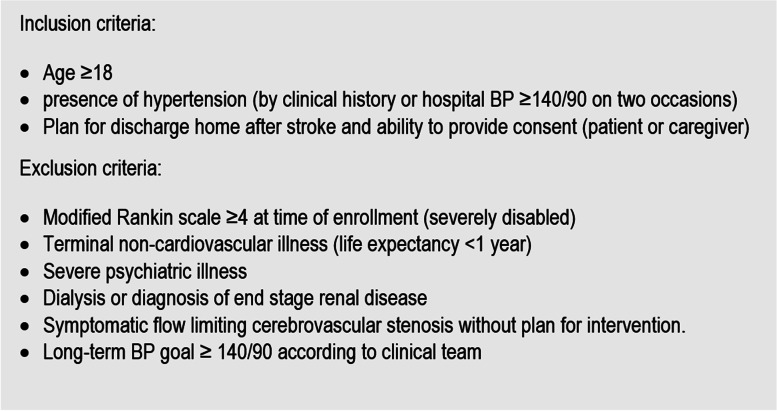
Eligibility criteria for TASC

### Patients who lack capacity

Many patients with stroke likely lack capacity to make decisions about their care. To be as inclusive as possible, we will include those patients who have caregivers but are able to respond to self-reported questions about their health. Capacity will be assessed during the screening process, and initial approach. If patients lack capacity, attempts will be made to identify and recruit an informal caregiver (such as family or friend) who can aid the patient, is involved in the medical decision-making process, and serves as the legally authorized representative. Such an individual will be noted in the informed consent.

### Trial timeline

The study starts recruitment after IRB approval, and each participant will be followed for 3 months from the time of acute stroke hospitalization setting in till three months post-acute stroke in the home setting (Fig. 1). Table 2 details the schedule of study enrollment, interventions, and assessments planned through the study till completion. The SPIRIT checklist which details the recommended items to include in a clinical trial protocol is available in Additional file 1.

**Table 2:**
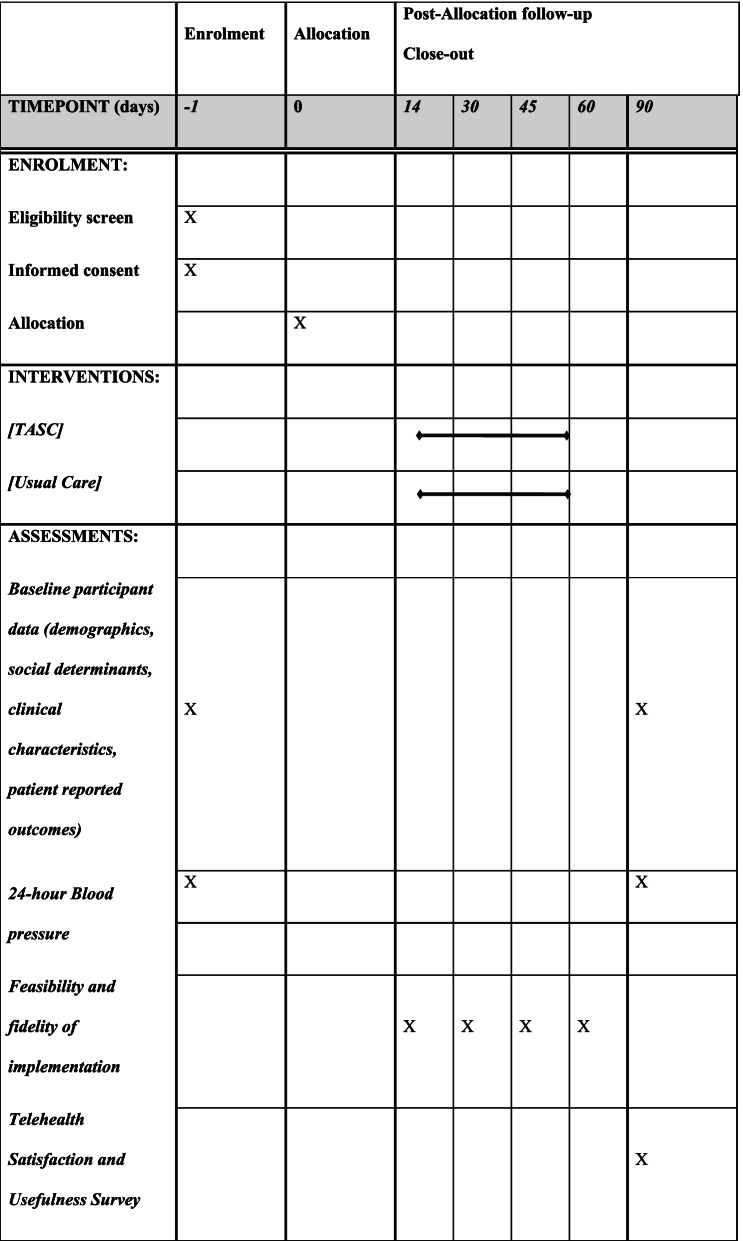
The schedule of enrolment, interventions, and assessments [as per Standard Protocol Items: Recommendations for Interventional Trials (SPIRIT)

### Screening and identification

Suitably qualified research staff and team members will screen and identify eligible patients from the stroke center admitted with acute stroke. All patients admitted with acute stroke will be screened for eligibility. To achieve adequate participant enrolment to reach target sample size, recruitment will be expanded to patients homebound from our inpatient acute rehabilitation if we are not able to meet recruitment targets. Detailed screening logs will record numbers of eligible patients, key reasons for ineligibility, and recruitment/refusal rates. Screening data will be used to complete the CONSORT diagram for interventional trials [62]. This will document the flow of participants through the study. Patients enrolled in other intervention trials will not approached without approval of principal investigators and with team consensus. Patients will continue to receive care through inpatient team and upon discharge, with outpatient providers at the patient’s discretion.

### Recruitment

Institutional Review Board (IRB) approval has been obtained for all processes. Recruitment is expected to take 6 months. To ensure a diverse patient population, bilingual research and clinical coordinators will be available as an integral part of the team for patient engagement at the time of enrollment. All instruments used will be available in English and Spanish versions for ease of use. Providers will be sensitive to need for translation services as applicable. Additional hospital-based interpretation services will be utilized on site on through telephone services if needed for Spanish speaking patients. Any participant requesting a willing family member to provide translation will also be included.

### Approach and consent

In addition to screening patients for inclusion and exclusion criteria, clinical team members will be consulted prior to approaching the patient (e.g., if they are well enough or available, not undergoing clinical testing and planned for discharge). Sequential eligible patients will be approached prior to discharge from the hospital to assess their interest in enrollment. The research team will provide verbal explanation of the study, and patients will have an opportunity to ask questions and consider their participation. If patients wish to take part, they will be provided with a written consent form to consider and complete. Legally authorized representatives (LARs) will provide consent for patients with aphasia who are otherwise interested. Baseline assessments of socioeconomic determinants of health and patient reported measures with validated surveys will be completed prior to participant randomization.

### Randomization and blinding

We will randomize patients (1:1) to intervention versus usual care using a computer-generated block design for parallel assignment. The randomization allocation sequence will be developed by a statistician not involved in the enrollment procedures. The outcomes assessors will be blinded after participant assignment to intervention We plan to stratify based on race to assure that the groups are balanced with respect to baseline risk and sample.

### Withdrawal

The rights of the patients to withdraw will be respected in the study. They will be informed of this in the consent process, and their care or treatment will not be affected. Data previously collected will be used in analysis and where possible, a reason for withdrawal will be recorded. If patients are unexpectedly discharged from the hospital to a nursing care facility, other than short period of acute rehabilitation stay, they will become ineligible and will be withdrawn.

#### Intervention group

##### TASC intervention program (components)

TASC program components of integrated care and multidisciplinary team approach are discussed as below and outlined in Table 3.

**Table 3:**
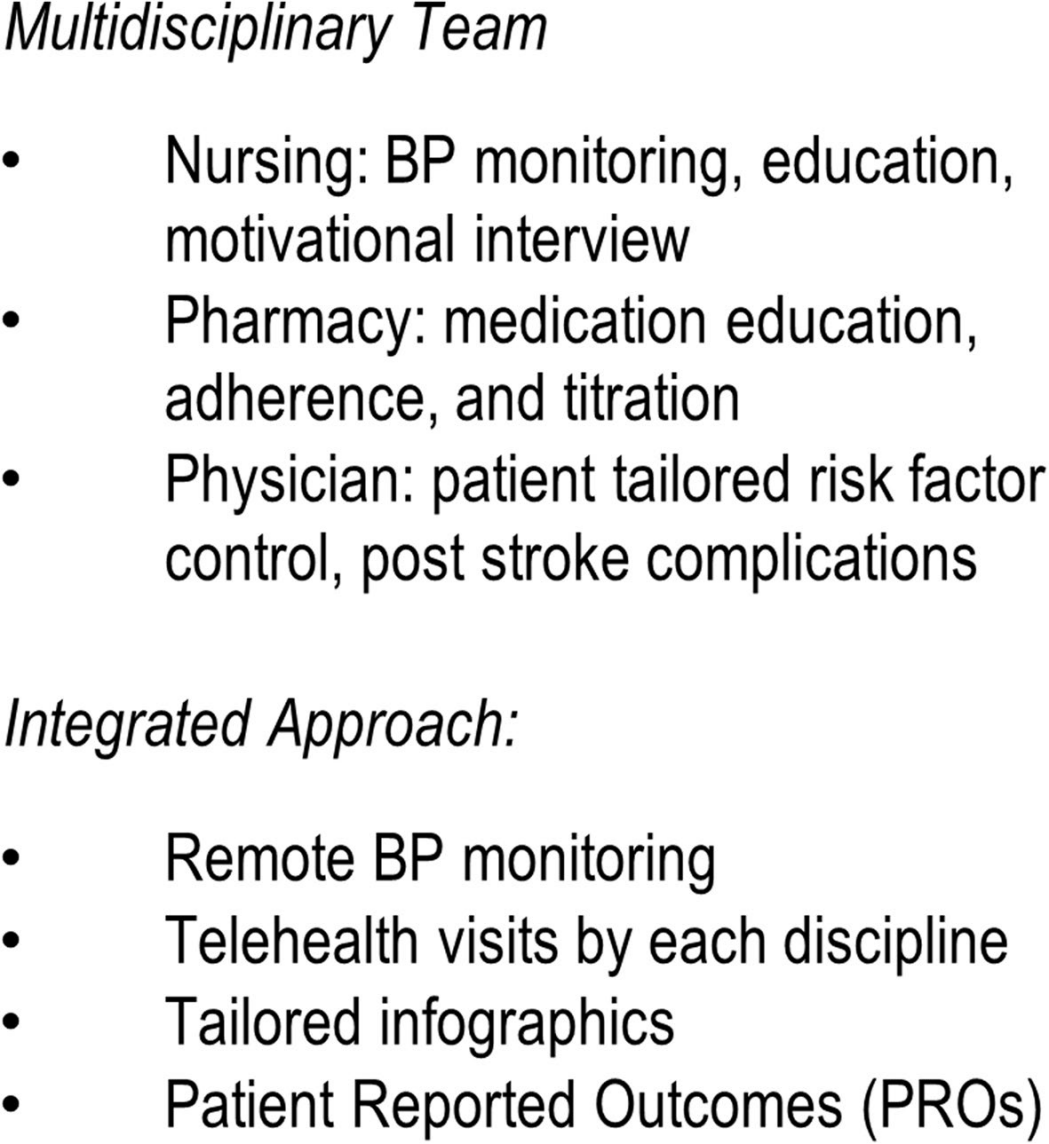
TASC components

##### Multidisciplinary team inclusive of a pharmacist

The newly assembled team consists of nursing, pharmacy, and physician care. This is a project that has brought together faculty from diverse disciplines who have not previously been involved as collaborators in post-stroke care.

A team providers meeting will be held bimonthly as a “share and learn” touch point. Patients undergoing follow up visits and remote BP monitoring will be discussed, issues identified, and an action plan generated to improve processes in care provided. Fidelity of implementation for each patient will also be reviewed with all providers. Any problems identified in providing the intervention, as well as mediators in enforcing follow ups and monitoring, will be used to inform further aims of the intervention.

Staff training will be provided to implement visually tailored infographics to patients regarding BP readings. Most recent recorded BP will be used to discuss BP goals and provide education regarding need for BP control. Remote BP monitoring team nurses will receive feedback regarding changes made to medication regimens at telehealth visits to provide guided support to patients at touch point calls. Staff schedulers and coordinators will work closely with providers and patients to schedule follow up appointments and coordinate patient care with updated contact information within discussed time frames. They will receive additional training in trial requirements and provide support for team members.

##### Remote BP monitoring

Our integrated care includes remote BP monitoring and electronic tablet devices provided through New York Presbyterian at no charge to patients with remote support including centralized telehealth nursing calls to patients for elevated readings and driven by patient feedback for frequency.

Patients randomized to telehealth services will be set up with a Philips Remote Blood Pressure Monitoring (RBPM) kit including an electronic device (Samsung tablet with 2-way video capable of telemedicine) and BP/pulse monitor and set up for remote monitoring. These services are designed to reduce disparities in technology literacy with home or remote installation as needed along with step-by-step instructions and explanations provided to each participant in their language of preference. Further, there is no need for patients to have their own technology or internet services as a tablet is included with data transfer capability. BP devices provided have been validated and calibrated for home use.

Certified nurses will touch base through phone calls with patients at intervals set to patient preference and escalate severely elevated BP readings to providers (Fig. 2). BP monitoring results as well as patient reported surveys completed on the electronic tablet are wirelessly transmitted to electronic health records and to a web-based platform (eCare Coordinator) available for the telehealth nurses and the patient’s treating clinical team to see. Critical BP or pulse readings will be shared via system alerts, at which point the call center nurse will contact the patient and assess. If unable, this is to be followed with escalation calls for elevated readings that require immediate attention to the TASC team for resolution (Fig. 2). If patients fail to measure BP data at home for a week that the device automatically transmits, they will be contacted by the call center nurse to enquire. If device trouble shooting is needed, technical assistance will be provided in person or remotely as indicated.

##### Telehealth visits by each discipline

**Fig. 3:**
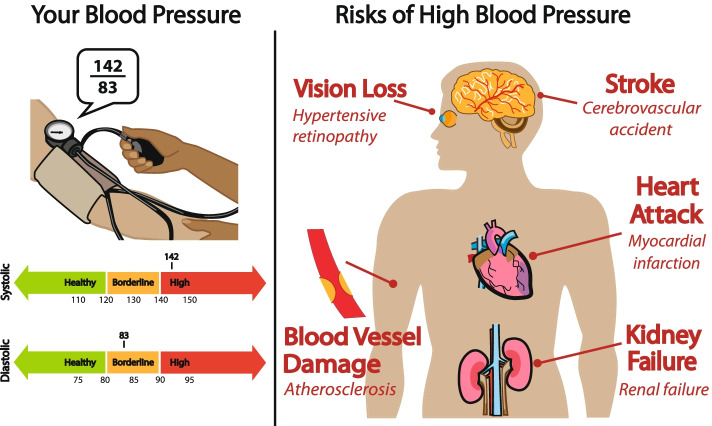
Tailored BP infographics used to conduct motivational interview regarding control

Patients/caregivers will receive instructions regarding equipment use for telehealth video visits and the first telehealth visits after discharge will be scheduled with the nurse practitioner at 1–2 weeks (± 5 days), pharmacist at 4 and 8 weeks (± 5 days), and physician at 6 and 12 weeks (± 5 days). These visits are set at different intervals to serve as different points of visual contact with the patient and reduce duration burden of each visit. At the first visit, patients will be shown on their tablet screen an infographic tailored with their most recent BP value at hospital discharge integrated into a transitions of care management visit with a primary care nurse practitioner (see Fig. 3). The infographic will serve as the centerpiece of a motivational interview about addressing barriers to medication adherence. The pharmacist will meet with patient to review medication adherence, effectiveness through BP log, and lifestyle modifications. When not well controlled and evidence-based guideline driven, the pharmacist will adjust medications under the collaborative drug therapy management agreement. Tele-pharmacy visits are possible through the collaborative drug therapy management law in New York State which allows eligible pharmacists to formulate agreements with physicians for chronic disease management [63]. They will also address side effects and interactions of medications at these visits. Treatment of hypertension and selection of BP lowering medications will be in accordance with latest published guidelines for BP management [64]. The physician will provide patient-tailored adjustments to the care plan and monitor for any adverse events, and stroke related complications.

##### Infographics

BP infographics (Fig. 3) are to be used to provide self-management awareness and BP visual education. These will be tailored to the patient’s blood pressure at the time of discharge and shared on the screen during the first telehealth visit to drive a motivational interview guided by patient responses regarding health behaviors [59].

##### Comparison group (usual care)

Participants randomized to comparison group will receive a one- and three-month post-stroke follow-up appointment. They will receive a transitional care management visit at 1-2 weeks post discharge in addition to stroke physician visits as part of the study to ensure timely follow ups. Although these may not be routinely possible, these are considered best practices. Patient-reported outcomes will be collected after consent and at three months follow-up including surveys and BP measurement.

### Study measures

Feasibility for the project will be monitored with metrics of at least one subject per week to be recruited, weekly and monthly recruitment rates assessed with estimates of these rates gathered at the end of the study. We will assess feasibility of randomization with expected targets of at least 70% of all eligible patients recruited during the enrollment period. We will also assess adherence and retention rates, with expected adherence of at least 70% to at least one video visit in each arm, and retention rates of at least 70% at study completion as stop-go criteria.

Since this is a pilot feasibility study, a sample size calculation was not performed. It is felt that *n* = 50 will be a large enough sample to inform about the practicalities of intervention delivery among patients returning home after an acute stroke. Specifically, with *n* = 25, there is about 85% power at 5% significance (one-sided) to demonstrate an adherence rate of 75% in an arm against a null rate of 50%. We note that in COMPASS-TC study [46] only 35% attended a clinic visit. Therefore, setting the null rate at 50% is reasonable.

Fidelity of implementation will be assessed using a brief checklist at each patient visit by each discipline. An internally designed and validated survey, Telemedicine Satisfaction and Usefulness Questionnaire (TSUQ) [65] provides measures of patient satisfaction and will be collected at 3 months among intervention patients to assess patient satisfaction with telehealth (Table 4). Self-Assessment of Interdisciplinary Research Competencies will be surveyed at the beginning and end of the project to establish progress and satisfaction among TASC providers and investigators in this collaboration [66–68].

**Table 4:**
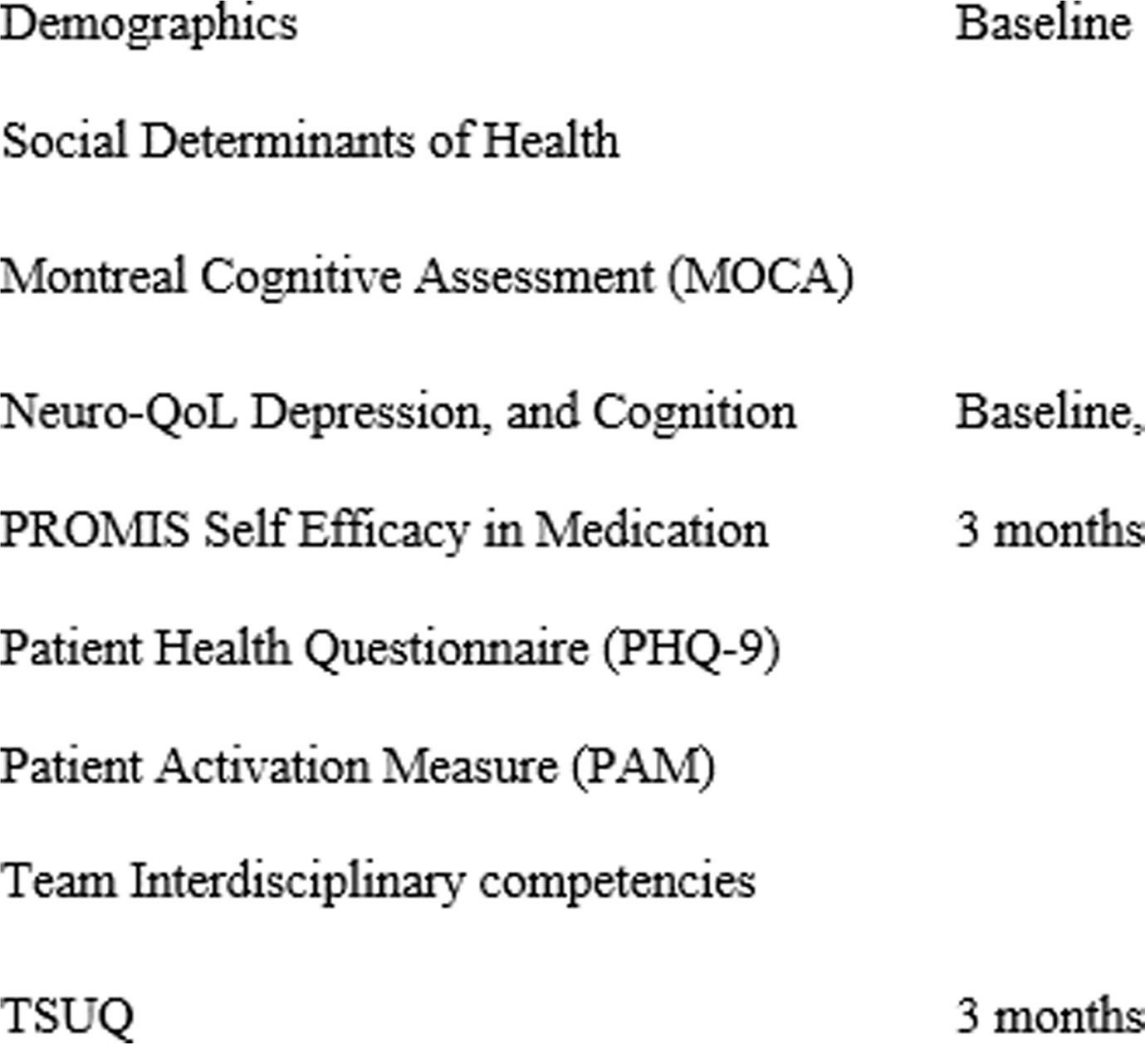
List of study measures

Patients will receive BP monitors at different times to meet ethical and clinical standards, either at the start for patients in the intervention arm or at study end in the usual care arm. RBPM will be conducted among participants in the intervention arm from time of discharge through to 3 months. Readings from the first 24 h at 3 months in control will be collected.

Patient-reported outcomes will be collected using the PROMIS tools (self-efficacy of medication adherence and treatment, Neuro-QOL Depression, and Cognitive Function) as well as patient activation measure (PAM) at time of consent and at 3 months among all participants. The effect of empowering stroke survivors’ self-management can be assessed through surveys of medication adherence and treatment self-efficacy, and activation in health behaviors.

### Data collection

Patient demographics, social determinants of health (age, education, race/ethnicity, address, income, insurance type, along with food, housing, and income security), internet and electronic devices access questions, and medical characteristics (risk factors, comorbidity burden, stroke adjudication) will be collected from the patient and electronic medical records in a data collection form at discharge and at 3 months.

RBPM will be set up for the intervention arm through to 3 months. Patients in the usual care arm will receive the BP monitoring kit with remote assistance at 11 weeks and provide readings at 3 months for outcome assessment. They will also be aided through the remote monitoring team with survey completion if needed. All completed surveys and BP readings will be converted to transferrable format and transmitted via the online platform to REDCap database at 3 months for study completion. Electronic medical records will also be revisited at study end for any additional surveys conducted during patient visits for clinical screening and management purposes.

### Data management, monitoring, and safety reporting

Patient data will be recorded on case report forms (CRFs) built into a REDCap (Research Electronic Data Capture) dictionary hosted at the Neurological Institute of New York, Columbia University. REDCap is a secure, web-based application designed to support data capture for research studies. Participants will be assigned a unique code identification number and all data will be completely anonymized for purpose of analysis and reporting. Electronic data and hard copies will be stored securely.

Data will also be monitored for quality and completeness. Investigators will do their utmost to ensure data completeness, and any missing data will be chased unless confirmed not to be available at the time of each patient close-out visit. Survey data will be directly collected from participants, and thus will not be subject to data verification. All consent forms will be encrypted and stored securely in the Neurological Institute. Any unexpected adverse effects will be reviewed by the primary investigator and reported to the sponsor and ethics committee.

### Data integration

Patient-reported outcomes (PROs) such as post-stroke depressive symptoms and cognitive function will be captured through web-based tablet-enabled software provided by Patient Reported Outcome Measurement Information System (PROMIS), developed by National Institutes of Health funded endeavors to provide precise and flexible electronically administrable tools. Surveys would also be uploaded to the tablet included in the RPBM kit for patients to complete and send automatically from the tablet. RBPM data and online surveys will be collected on a web-based platform and transferred in configured format to integrate into REDCap as patients complete the study. Telehealth visit data will be collected from electronic medical records. The video platform utilized will be noted, to ensure that only currently available HIPAA compliant applications are used.

### Statistical analysis

As this is a feasibility study with a small sample size, pilot data will be used to assess feasibility of the intervention and estimate the variability in the endpoint, while provide preliminary evidence for BP control. A subsequent trial designed will be powered to detect the clinically significant difference to plan a larger confirmatory trial. However, more importantly, descriptive statistics will characterize the randomized patients completing surveys and outcome assessments. Generalized linear modeling will evaluate systolic BP outcome 90 days post discharge as a function of treatment, race/ethnicity, and the interaction of treatment arm and race/ethnicity. We will explore moderating effects of race/ethnicity as secondary analysis. Decisions regarding the pursuit of a subsequent trial will be based on feasibility of the trial, and analysis of all other measures will be hypothesis generating.

## Discussion

Improvement in acute care treatments and prevention have led to decreased mortality among stroke survivors, taking stroke from the third to the fifth leading cause of death in the USA. Moreover, between 2010 and 2030, stroke is projected to have the largest relative increase in total direct medical costs of 238% reaching $95.6 billion and total indirect medical costs of 73% reaching $44.4 billion in the USA [69]. About one-half of patients who survive a stroke are at increased risk of recurrent stroke within a few days or weeks of the initial event, with the greatest risk during the first week [70]. In terms of health care utilization, readmission hospitalizations cost greater than $17 trillion dollars for Medicare [71] and result in hospital penalties. Readmission rates from national data sets suggest up to 21% to occur within the first 30 days after a stroke, with about 1 out of 8 readmissions being considered preventable [72, 73]. The post-acute stroke period offers a valuable target for focused attention to improve patient-centered outcomes.

This pilot study will serve to promote our understanding of the gaps in our population for post stroke care. These data will help us to determine the feasibility of the TASC approach and collect preliminary data for further studies to develop effective telehealth based post-stroke systems of care. Cost-effectiveness analysis of TASC approach will help us understand health care utilization and will also provide insight for a sustainable secondary stroke prevention framework while addressing health disparities.

If feasibility of the TASC multidisciplinary is established, an adequately powered efficacy trial will be planned with goal to study cost-effectiveness of this approach in addition to adequate BP control with enhancement in self-management. In terms of BP telemonitoring, value-based purchasing of equipment may support team-based support. Hospitals may be incentivized with cost savings due to decreased hospital readmissions that are not reimbursed by public insurance. Further, downstream revenue generation in terms of referrals and appropriate testing can also be assessed. These resources can be utilized back among those with limited access to care to selectively provide support among targeted highest risk groups.

### Implementation and future application

The benefits of this study include identifying efficient and effective post stroke care systems, improving access to meaningful care, reducing the risk of recurrent stroke, and promoting patient-centered care. Feasibility of the intervention will be assessed to identify barriers and enablers to complete multidisciplinary telehealth visits and remote monitoring. Key potential problems are difficulties with recruitment and non-return of participants. Barriers to follow up will be addressed by appointment coordination and reminders, as well as coverage of travel costs if needed. If intervention arm participants have technical difficulties, staff will have capacity to make a home visit.

Pilot data collected will also enable our collaborative multidisciplinary team to refine the intervention. Data collected will eventually serve as feasibility data for subsequent study with a primary outcome of within-patient change in systolic BP, and for further peer-to-peer community-based interventions advancing health information.

An additional long-term aim of this project is to develop a long-standing collaboration among researchers in neurology, pharmacy, nursing, epidemiology, and biostatistics to develop additional studies that explore the intersection of community health in our disparate population. It will also allow us to explore successful clinical trial methodology for further implementation in current health systems.

## Conclusion

Based on this study, we will be able to develop further studies that solidify research collaborations among investigators from different disciplines around a common interest in the intersection of post-stroke care. The data will provide feasibility of the TASC approach, and its application through a telehealth based platform to advance team science, and improve stroke outcomes.

## Supplementary Information

**Additional file 1.** SPIRIT checklist.

## Data Availability

Requests for access should be made to the corresponding author and may be considered on a case-by-case basis by the Investigator group. All data requests for quantitative data will be managed in accordance with Columbia University processes and procedures.
